# Generic Versus Branded Atorvastatin: A Single-Center Comparative Analysis of Clinical Effectiveness and Post-Delisting Cost Savings in Saudi Arabia

**DOI:** 10.3390/healthcare14182983

**Published:** 2026-09-12

**Authors:** Abdulmajeed Hassan Jaafari, Abdulaziz Sami Al-Shwairkh, Miteb A. Alanazi, Abdullah M. Alhammad, Yazed Alruthia

**Affiliations:** 1 Department of Clinical Pharmacy, College of Pharmacy, King Saud University, Riyadh 11451, Saudi Arabia; 2 Corporate Department of Pharmacy, King Saud University Medical City, Riyadh 12372, Saudi Arabia

**Keywords:** atorvastatin, generic, effectiveness, dyslipidemia, cost savings, cholesterol, LDL

## Abstract

**Background:** Dyslipidemia is becoming an increasingly important issue from a public health point of view and is generally treated with statins. However, as generic versions of these drugs are now widely available, doubts have emerged about their effectiveness. The current study examines the clinical effectiveness and impact on the national budget of using generic atorvastatin compared to the branded version. **Methods:** A retrospective comparative study was conducted at King Khalid University Hospital between 2015 and 2025, involving 286 patients (142 receiving the generic form and 144 receiving the branded form). Changes in lipid panel values were assessed using a Generalized Linear Model (GLM) to account for multiple factors. The economic outcomes were estimated using probabilistic (10,000 Monte Carlo iterations) and deterministic scenario analyses. **Results:** The mean adjusted decrease in low-density lipoprotein (LDL) was 26.11% in the generic group and 19.34% in the branded group. After adjusting for multiple variables, the branded group showed a non-significant lower reduction in LDL (β-estimate: −0.255, 95% CI: −0.119 to 0.391, *p* = 0.3318). The Monte Carlo simulations showed large projected mean annual savings when using the generic formulation: $29,717,778 based on tender pricing and $295,432,159 based on retail pricing. The deterministic sensitivity analyses consistently found positive projected savings across all population and market-share scenarios tested. **Conclusions:** Generic atorvastatin was found to have similar clinical effectiveness to the branded version in terms of reducing atherogenic lipids, with no statistically significant difference between the two groups. Furthermore, the economic modeling shows that using generic atorvastatin yields considerable national-level cost savings.

## 1. Introduction

Dyslipidemia, which is characterized by abnormal concentrations of lipids such as low-density lipoprotein (LDL), high-density lipoprotein (HDL), and triglycerides, is a major modifiable risk factor for cardiovascular disease (CVD) [1]. Worldwide and especially in the Middle East and in Saudi Arabia, CVD remains the leading cause of illness and death [2]. In Saudi Arabia, the prevalence of dyslipidemia has always been between 20% and 40% [3,4]. Recent epidemiological data from 2023 show that the age-standardized prevalence of dyslipidemia is still high, going above 31% in the general adult population and increasing even more in people who have comorbid type 2 diabetes [4]. The main modifiable risk factors are unhealthy lifestyle habits, diabetes mellitus, hypertension, and unemployment, while older age and male sex are the main non-modifiable risk factors [5]. Apart from its clinical effects, dyslipidemia also places a heavy economic burden on healthcare systems around the world. In the United States, annual health expenditures for the management of dyslipidemia range from $17 million to $259 million [6]. Although there are few comprehensive cost-of-illness studies available in Saudi Arabia, the continuously high prevalence of dyslipidemia probably causes a considerable financial strain on the national healthcare system.

The use of medication, especially that involving statins, is still the main method for treating dyslipidemia and for reducing cardiovascular risk [7]. The important clinical effects of statins were demonstrated in major studies such as the Scandinavian Simvastatin Survival Study (4S), which reported a 30% reduction in overall mortality in high-risk patients with coronary heart disease (CHD) receiving secondary prevention [8,9]. The West of Scotland Coronary Prevention Study (WOSCOPS) then showed a 31% reduction in CHD cases over a five-year period in the context of primary prevention [10]. Further large-scale trials have repeatedly confirmed the ability of statins to lower LDL-C levels [11]. This strong body of evidence is shown in the 2026 American Heart Association/American College of Cardiology (AHA/ACC) Guidelines on the Management of Dyslipidemia and in the 2025 European Society of Cardiology (ESC) Focused Updates, both of which state that high-intensity statins—for example, atorvastatin—should be the first-line treatment for both primary and secondary CVD prevention [12,13]. Even though other drugs such as ezetimibe are available for patients who need stricter control of their lipid levels, atorvastatin is still the main therapeutic choice.

As dyslipidemia becomes more common, it is important to address its economic impact through cost-effective measures. Since generic statins are available, this offers a practical way to reduce healthcare costs without compromising therapeutic effectiveness. Nevertheless, there is controversy over the substitution of brand-name drugs with generics, particularly regarding their comparative safety and effectiveness in clinical practice. Surveys show that a large number of healthcare professionals are still skeptical; about one-third of doctors doubt the quality, safety, and therapeutic equivalence of generic formulations [14,15].

On the other hand, clinical evidence relating to these issues shows reassuring results. Large-scale database and cohort studies usually confirm comparable clinical outcomes for generic drugs [16,17]. A thorough comparative effectiveness study showed that both generic and branded medications lead to similar outcomes, with differences mainly attributable to perception bias or the ‘nocebo’ effect [16]. Moreover, a large cohort study that compared the start of treatment with generic versus branded statins found that patients who were prescribed the generics had an 8% lower rate of hospitalization for acute coronary syndrome, stroke, or all-cause mortality; this result was mainly caused by better medication adherence as a result of lower out-of-pocket expenses [17]. Cost-effectiveness analyses of atorvastatin have shown that the generic form provides considerable economic value across various clinical subgroups [18].

The extent to which generic drugs are used has a major effect on national drug prices, although adoption levels vary across regions [19]. In Europe, the use of generics varies greatly, ranging from 17% in Switzerland to more than 80% in the United Kingdom, and this is inversely related to the prices of widely prescribed drugs such as atorvastatin [19]. In Saudi Arabia, generic medicines previously had a smaller market share (about 35% in 2019) [20]. Nevertheless, as part of the healthcare transformation element of Saudi Vision 2030, the National Unified Procurement Company (NUPCO) was established to consolidate and streamline pharmaceutical purchasing across both the Ministry of Health and the military sectors [20]. To achieve financial viability, the most recent centralized procurement systems have clearly shifted toward local pharmaceutical production and prioritized cost-effective generics [21,22]. As a result, several expensive, imported branded drugs—including the branded atorvastatin—have been strategically withdrawn from and removed from the market when adequate generic alternatives are available [23].

Even with such proactive national initiatives, there remains a serious shortage of local, real-world data on the clinical and economic effects of switching to generic drugs. It is important to ensure that generic atorvastatin has an observed effectiveness comparable to the brand-name drug, especially for high-risk patients, so that economic benefits do not come at the expense of cardiovascular outcomes. For this reason, the present study seeks to examine the clinical effectiveness and the budget impact at the national level of generic atorvastatin as compared with brand-name atorvastatin after the brand-name formulation has been withdrawn and delisted in Saudi Arabia.

## 2. Materials and Methods

### 2.1. Study Design and Setting

The study employed a retrospective, observational approach based on a review of medical charts together with a budget impact analysis. The clinical data were obtained from the electronic medical records (EMRs) of patients who visited the Primary Care Outpatient Clinics and the Outpatient Cardiology Clinics at King Khalid University Hospital (KKUH) in Riyadh, Saudi Arabia. The data collection spanned a ten-year period, from 2015 to 2025. The study focused on patients who had been on continuous and uninterrupted statin therapy for at least 12 months, defined as those with active prescription renewals and pharmacy refill records in the EMR without any gaps, formulation changes, or dose modifications. To evaluate patients’ response to treatment, longitudinal low-density lipoprotein (LDL) levels were measured at baseline and at 3-, 6-, 9-, and 12-month intervals, with the 12-month measurement used as the primary follow-up point for comparison. The quarterly checks of the EMR were carried out merely to verify that the patients had continuous medication possession and were not used for any interim laboratory assessments.

The ten-year period during which the data were extracted was deliberately designed to include patient data from two different policy phases. The central process of withdrawing and removing the branded atorvastatin began in 2019; therefore, the patients assigned to the branded group are mainly those who received treatment between 2015 and 2019, while the generic cohort consists of patients treated under the generic requirement from 2019 to 2025.

### 2.2. Study Population and Eligibility Criteria

The group being studied consisted of adult patients who had been diagnosed with secondary (acquired) dyslipidemia. To avoid confounding by other lipid-lowering treatments, the study included only patients receiving atorvastatin monotherapy. These patients were divided into two groups: those taking the branded version and those taking the generic version.

Inclusion Criteria:

- People aged 18 or older.
- Individuals who have been diagnosed with secondary (acquired) dyslipidemia for at least 12 months.
- It was either kept as a strictly generic or a strictly branded atorvastatin for a period of at least 12 months (patients who had their dosage changed or who crossed over from a generic to a branded formulation during the 12-month period were not eligible).
- At the start of the study, the patient was statin-naïve (meaning that there was no prior record of having taken atorvastatin or any other statin at any time within the EMR of King Khalid University Hospital before the date on which the treatment was first started).

Exclusion Criteria:

- Patients diagnosed with familial dyslipidemia.
- Patients who stopped taking statins or who received treatment for a period of less than 12 months.
- Patients who are using other therapies together for dyslipidemia, such as PCSK-9 inhibitors, fibrates, or other non-statin lipid-lowering agents.
- Patients who had previously received treatment with statins or other therapies for dyslipidemia before the current prescription.
- Those patients who had missed either the baseline or the 12-month follow-up comprehensive lipid panels were included in the complete case analysis for the evaluation of the primary endpoint.
- Patients who, at any time during the 12-month follow-up period, had their dose of index atorvastatin increased or decreased.
- Patients who changed their treatment from the branded to the generic atorvastatin during the 12-month follow-up period were excluded to ensure that the clinical outcomes clearly showed results for only one formulation.

### 2.3. Sample Size Estimation

The minimum required sample size was calculated before data extraction to ensure adequate statistical power for comparative analysis. Using an alpha level of α = 0.05, a beta level of β = 0.20 (resulting in 80% power), an expected effect size of Cohen’s d = 0.3, and a 1:1 allocation ratio between the branded and generic cohorts, the minimum total sample size was estimated at 278 patients.

### 2.4. Study Variables and Data Collection

Data were systematically collected from the KKUH electronic medical records (EMRs). The original raw data were entered into customized Excel spreadsheets and encrypted to protect them against unauthorized access or breaches. To achieve the highest standard of data integrity and prevent extraction errors, a separate, independent audit was conducted on the final dataset, systematically checking all baseline and 12-month follow-up lipid values against the original electronic medical records before statistical modeling.

The variables collected included:

- **Demographics and Anthropometrics:** age, gender, weight, height, and BMI.
- **Clinical Characteristics:** number and types of medical comorbidities, and the total count of active medications (defined as the total number of unique pharmacological substances prescribed at baseline).
- **Pharmacological Data:** atorvastatin type (generic vs. branded) and daily dose prescribed.
- **Laboratory Outcomes:** lipid profiles (LDL, TC, TG, and HDL) measured at two specific points, baseline (just before the index prescription) and at 12-month follow-up. Intermediate lab values were not routinely collected, aligning with standard annual monitoring for stable patients.
- **Clinical Outcome Measures:** absolute and percentage changes in lipid parameters (LDL, TC, TG, and HDL). These were calculated by comparing baseline laboratory values, measured just before the initial prescription, with final laboratory values recorded at the end of the 12-month continuous therapy. Lipid measurements taken before the 12-month endpoint were excluded from these final values.
- **Economic Data:** unit prices for atorvastatin formulations sourced from the publicly available Saudi Food and Drug Authority (SFDA) database for retail prices. As tender prices are proprietary, bounds were determined from historical purchasing contracts (2019–2025) in the King Khalid University Hospital (KKUH) electronic supply chain. These contract bounds were then verified by senior hospital procurement pharmacists to confirm they align with typical national procurement tiers.

### 2.5. Ethical Considerations

The study adhered fully to the ethical principles outlined in the Declaration of Helsinki. All patient data were completely de-identified, and only the principal investigators were granted access to the encrypted dataset, which was maintained in a secure digital environment. The Research Ethics Committee of the King Saud University College of Medicine in Riyadh, Saudi Arabia, reviewed and approved the study protocol (Approval Reference: Research Project No. E-25-9555).

### 2.6. Statistical and Economic Analysis

All statistical and economic analyses were performed utilizing SAS version 9.4 (SAS Institute, Cary, NC, USA). The economic modeling was conducted from two perspectives: the public healthcare payer perspective (using centralized public-sector tender pricing) and a broader societal/private payer perspective (using retail pharmacy pricing to capture patient out-of-pocket costs and private insurance impacts).

#### 2.6.1. Clinical Effectiveness Analysis

The study used descriptive statistics to summarize baseline demographic and clinical features. For continuous variables, the mean and standard deviation (SD) were reported, and for categorical variables, the frequencies and percentages were provided. To make inferential comparisons between the generic and branded groups, t-tests were applied to continuous variables, and Chi-square or Fisher’s exact tests were used for categorical variables, depending on the situation. A complete-case analysis was carried out for the main baseline and 12-month laboratory outcomes, and missing intermediate lipid measurements (at 3, 6, or 9 months) were not statistically imputed, since they were mainly used as observational measures of adherence rather than as inputs for the generalized linear models.

To examine comparative clinical effectiveness, multiple linear regression was used to assess the effect of generic versus branded atorvastatin on the percentage decrease in LDL levels from baseline to the 12-month follow-up. Generalized linear models were used to compare adjusted mean percentage reductions in LDL, TG, and TC, with a normal distribution and a log link. Before interpreting the results, strict diagnostic procedures were assessed for key statistical assumptions. The normality of the model residuals was assessed using Q-Q plots, and homoscedasticity was assessed using scatterplots of residuals against fitted values. To ensure that standard errors were not artificially inflated, multicollinearity among the adjusted clinical covariates was evaluated using Variance Inflation Factors (VIFs), and all variables were required to have VIFs strictly below 5. Lastly, the model’s overall fit and simplicity were evaluated using the Akaike Information Criterion (AIC). These GLM analyses accounted for potential covariates and baseline imbalances, including patient age, gender, statin dosage (modeled as a continuous variable in milligrams to improve precision in adjusting for baseline dose imbalances), BMI, the number of comorbidities, and the total number of concurrent prescription medications. Since the branded and generic groups were primarily treated in sequential time periods (pre-2019 and post-2019, respectively), these GLM analyses were essential to reduce temporal confounding. By accounting for baseline covariates that may have changed over the ten-year study period, the models aimed to better estimate the comparative clinical effect of the atorvastatin formulations, though we acknowledge that residual and temporal confounding cannot be entirely eliminated. Moreover, a GLM was selected rather than a longitudinal repeated-measures model to focus on the clear, long-term therapeutic response after a full 12 months of continuous treatment. This specific comparison intentionally matches the clinical endpoint with the annual timeframes used in economic modeling.

#### 2.6.2. Budget Impact and Cost-Savings Simulation

A budget impact model was created to compare national spending on atorvastatin and estimate potential savings after removing branded atorvastatin from the market from 2019 to 2025. To address uncertainties in market conditions and pricing, probabilistic sensitivity analyses were performed using Monte Carlo simulations with 10,000 iterations. These simulations used probability distributions tailored to each parameter type to capture empirical uncertainties. Epidemiological data, such as dyslipidemia prevalence (3.4% to 6.9%) and market share (38.0% to 86.1%), were modeled with Beta distributions. Economic variables, including the minimum and maximum unit prices for generic and branded drugs, were modeled with Gamma-uniform distributions to reflect public tender tiers and retail pricing. To keep estimates conservative given limited local correlation data, all parameters were simulated as independent, with no predefined correlations. A Deterministic Scenario Matrix was also developed to project exact annual costs and differences based on base-case prices.

The simulation utilized the following primary parameters:

- **Population and Market Share:** While the epidemiological prevalence of dyslipidemia exceeds 31%, the economic model utilized the diagnosed and pharmacologically treated prevalence to accurately reflect actual healthcare utilization. According to data synthesized from the National Platform for Health and Insurance Exchange Services (NPHIES) [24], the Saudi General Authority for Statistics [25,26], and the published literature estimates [3,4,5,27,28,29], the treated prevalence among the adult population in Saudi Arabia as of 2024 ranges from 3.4% to 6.9% (specifically reported at 3.4%, 3.6%, 4.4%, and 6.9%). Based on these treated prevalence rates (3.4%, 3.6%, 4.4%, and 6.9%), the total populations eligible for and actively using statin therapy are estimated at 941,105, 985,441, 1,204,428, and 1,877,812 individuals, respectively. Crucially, the model does not assume all these individuals receive atorvastatin; rather, the specific market share of atorvastatin among this pharmacologically treated class was subsequently applied across three distinct utilization scenarios (38.0%, 52.9%, and 86.1%) to isolate the final target population [29,30,31].
- **Generic Pricing Structure:** Public tender unit prices (representing the centralized procurement cost to the public healthcare payer) ranged from USD 0.033 to USD 0.06506 per tablet. Retail pharmacy unit prices (representing the cost to private insurers or out-of-pocket patients) ranged across multiple tiers (USD 0.293, USD 0.386, USD 0.5413, and USD 0.728).
- **Branded Pricing Structure:** Public tender unit prices (cost to the public payer) ranged from USD 0.114 to USD 0.2038 per tablet. Retail pharmacy unit prices (cost to private payers or patients) ranged from USD 1.114 to USD 2.038.

## 3. Results

### 3.1. Baseline Characteristics of the Study Population

A total of 286 patients met the inclusion criteria and were included in the analysis, with 142 (49.6%) receiving generic atorvastatin and 144 (50.4%) receiving the branded formulation. As detailed in Table 1, the mean age of the overall cohort was 57.16 ± 9.75 years, and no statistically significant difference in age was observed between the generic (56.25 ± 10.18 years) and branded (58.06 ± 9.26 years) groups (*p* = 0.1169). The cohort was predominantly female (57.69%), with an equal gender distribution between the two treatment arms (*p* = 0.9853). Baseline Body Mass Index (BMI) was also comparable between the generic (30.84 ± 5.91) and branded (31.26 ± 6.02) cohorts (*p* = 0.5425).

While demographics were largely balanced, statistically significant differences were observed in specific clinical parameters and treatment regimens (Table 1):

- Patients in the branded atorvastatin cohort were prescribed a significantly higher mean number of concurrent active medications (6.92 ± 3.74) compared to the generic cohort (5.46 ± 2.88) (*p* = 0.0002).
- Hypertension was significantly more prevalent among patients receiving the branded formulation (60.42%) compared to those receiving the generic version (48.59%) (*p* = 0.0446).
- A significant variation was noted in the prescribed atorvastatin dosages (*p* = 0.0012); a higher proportion of generic users were maintained on the 10 mg dose (46.48% vs. 33.33%), whereas branded users were more frequently prescribed the 40 mg dose (22.22% vs. 7.75%).

### 3.2. Clinical Effectiveness and Lipid Panel Outcomes

Both the generic and branded atorvastatin formulations demonstrated highly significant efficacy, achieving substantial, favorable reductions across all atherogenic lipid parameters, specifically low-density lipoprotein (LDL), triglycerides (TG), and total cholesterol (TC), from baseline to the 12-month follow-up period (*p* < 0.0001). However, a modest, non-significant increase in HDL was observed across both groups. As summarized in Table 2, patients treated with generic atorvastatin saw mean low-density lipoprotein (LDL) levels decrease from 3.46 ± 0.96 mmol/L to 2.26 ± 0.58 mmol/L, resulting in a mean absolute reduction of 1.20 ± 0.81 mmol/L. Similarly, the branded cohort’s mean LDL decreased from 3.22 ± 0.96 mmol/L to 2.36 ± 0.63 mmol/L, for a mean reduction of 0.86 ± 0.84 mmol/L.

**Figure 1:**
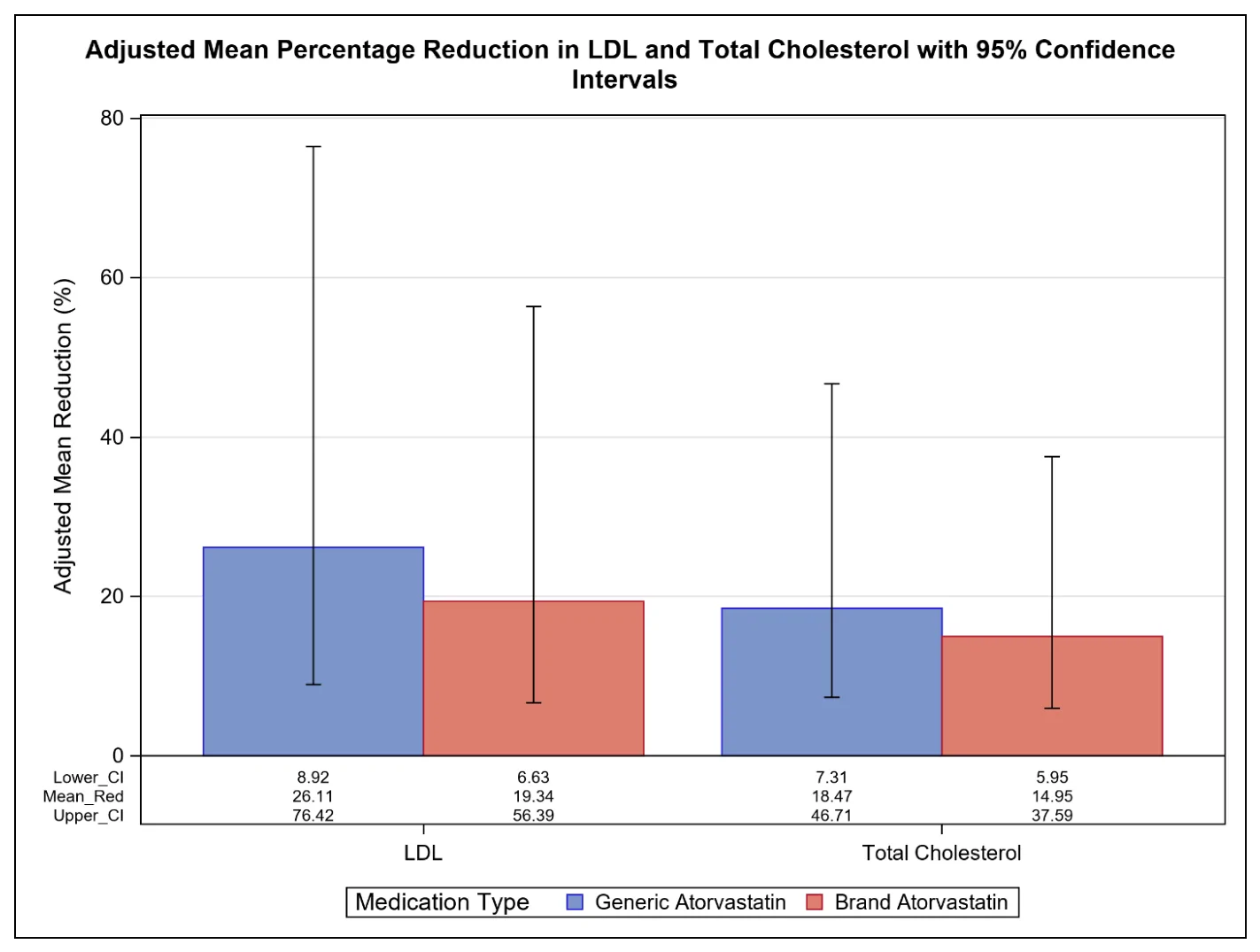
Adjusted mean percentage reductions in LDL and Total Cholesterol (TC) among patients on generic versus branded atorvastatin.

Parallel statistically significant reductions were observed in Triglycerides (TG) and Total Cholesterol (TC). Patients on generic atorvastatin had a mean reduction in TC of 1.27 ± 0.92 mmol/L, whereas the branded group had a mean reduction of 0.95 ± 0.93 mmol/L. Reductions in TG were comparable, with decreases of 0.20 ± 0.49 mmol/L and 0.16 ± 0.55 mmol/L for the generic and branded groups, respectively. Conversely, HDL levels remained clinically stable, with a slight expected increase, rising from 1.36 ± 0.38 to 1.38 ± 0.37 mmol/L in the generic group (*p* = 0.7129) and from 1.22 ± 0.36 to 1.23 ± 0.33 mmol/L in the branded group (*p* = 0.1864). The adjusted mean percentage reductions in LDL and TC, with 95% confidence intervals, are shown in Figure 1.

### 3.3. Factors Influencing LDL Reduction

To evaluate the specific impact of the atorvastatin formulation on LDL levels while controlling for confounding clinical variables, a multiple linear regression analysis was conducted (Table 3). The model revealed that the choice between branded and generic atorvastatin was not a statistically significant independent predictor of LDL reduction (β-estimate = −0.255; 95% CI: −0.119 to 0.391; *p* = 0.3318).

Among all the evaluated covariates, only Baseline LDL emerged as a statistically significant predictor influencing LDL levels (β-estimate = −0.660; 95% CI: −0.726 to −0.594; *p* < 0.0001). Unlike the previous iteration of the model, the total number of prescription medications was no longer significant after adjusting for baseline LDL (*p* = 0.1890). Other patient factors, including age (*p* = 0.6871), gender (*p* = 0.4142), continuous statin dosage in milligrams (*p* = 0.3814), number of baseline comorbidities (*p* = 0.1674), and baseline BMI (*p* = 0.4710), did not reach statistical significance within the model.

### 3.4. Budget Impact and Cost-Savings Analysis

The economic impact of using generic versus branded atorvastatin was quantified using 10,000 Monte Carlo simulations; the results are presented in Table 4. The simulations indicated substantial projected national cost savings associated with the generic formulation. Under public tender pricing structures, the estimated mean annual cost of procuring generic atorvastatin was $13,321,182 (95% CI: $13,186,441 to $13,455,924), in sharp contrast to $43,038,960 (95% CI: $42,613,772 to $43,464,149) for the branded equivalent. This price differential yields a mean annual projected tender savings of $29,717,778.

When evaluating expenditures based on retail pharmacy pricing, the financial disparity widened significantly. The simulated mean annual retail cost for branded atorvastatin reached $427,258,865, compared to $131,826,706 for the generic counterpart. Consequently, transitioning to generic atorvastatin results in an estimated mean projected annual retail savings of $295,432,159 (95% CI: $292,146,122 to $298,718,197).

### 3.5. Deterministic Scenario and Sensitivity Analyses

**Figure 2:**
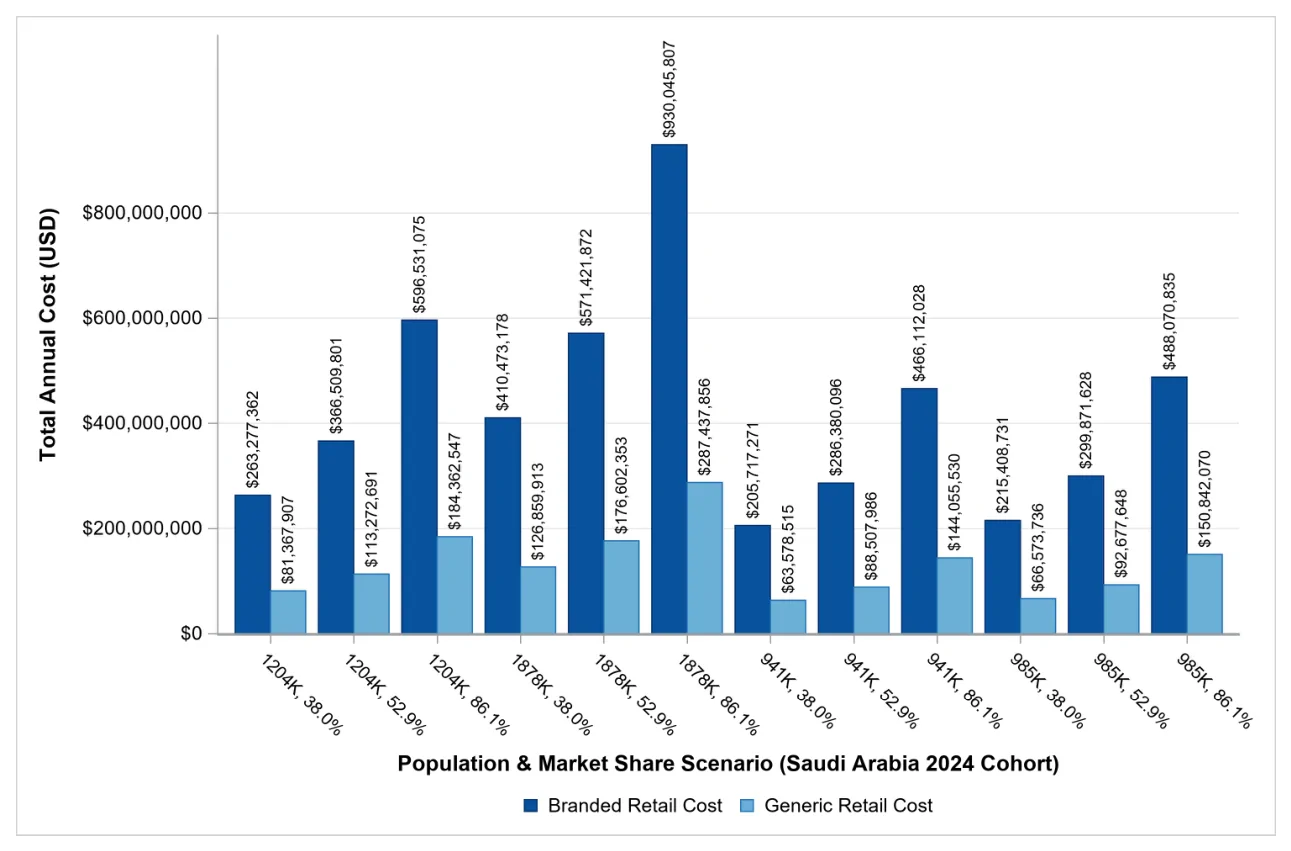
Scenario Analysis of Total Annual Retail Costs Across Varying Population and Market Share Estimates: Generic vs. Branded Atorvastatin.

A deterministic scenario matrix (Table 5) was utilized to project exact annual costs and calculate differences across varying demographic thresholds and market share estimates using base-case pricing. In the most conservative scenario, assuming the lowest eligible population estimate of 941,105 and a 38.0% market share, the projected annual retail savings still amounted to $142,138,756, with tender savings projected at $14,341,470. In the maximum utilization scenario (a population of 1,877,812 with an 86.1% market share), total projected annual retail savings peaked at $642,607,951, while projected tender savings reached $64,837,648. A comparative visual analysis of these varying population and market share scenarios on total annual retail costs is provided in Figure 2.

**Figure 3:**
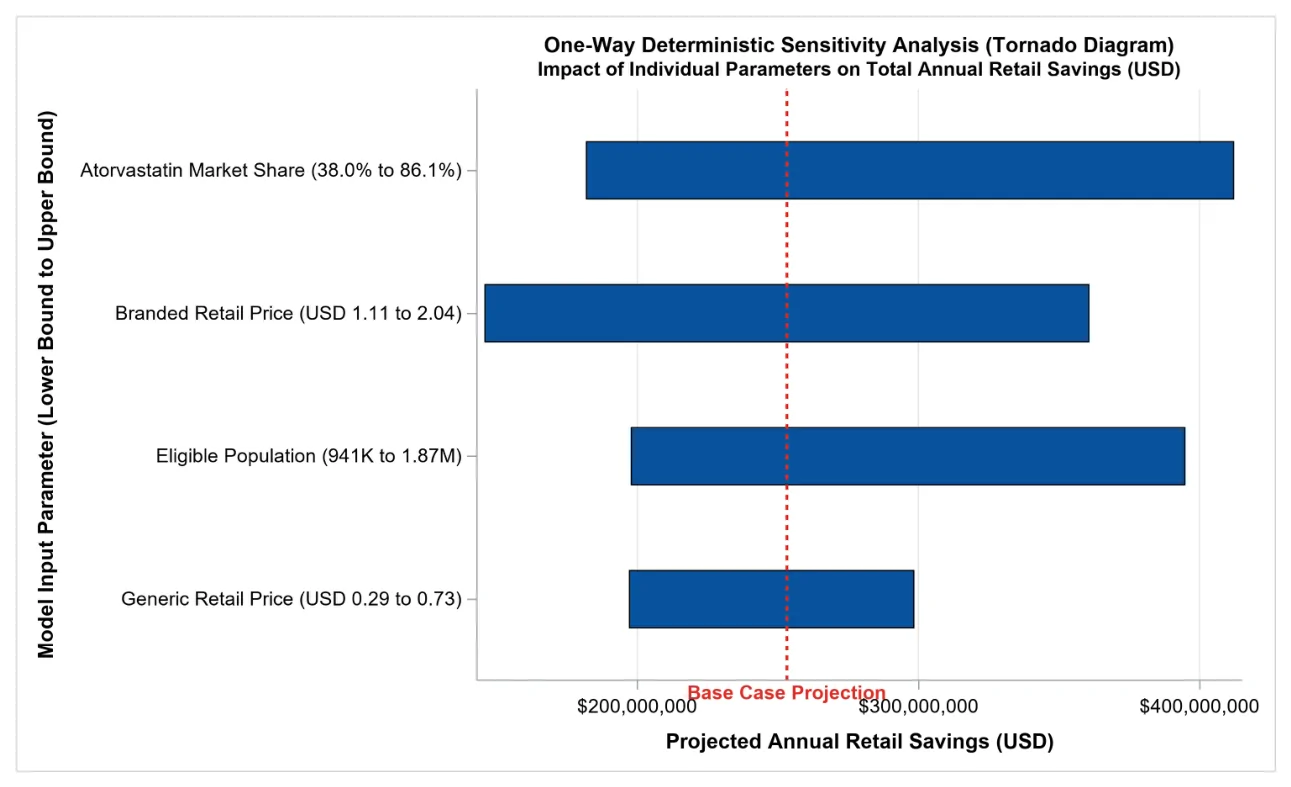
Sensitivity Analysis of Input Parameters Driving Total Annual Retail Cost Savings.

The one-way deterministic sensitivity analysis (Figure 3) shows how changes in individual model parameters affect the projected annual retail savings, which are about $250 million. The market share of atorvastatin has the greatest effect on economic variability; increasing the use of the generic drug from 38.0% to 86.1% would raise potential savings from about $180 million to more than $400 million. Both the current branded retail price and the total number of eligible patients significantly influence baseline cost avoidance. On the other hand, variations in the generic retail price (ranging from $0.29 to $0.73) have the smallest impact on the economic model. Even in the most conservative scenarios, such as assuming the highest possible generic price and the lowest level of market penetration, the model still forecasts national annual savings exceeding $150 million, confirming the strong financial benefit of replacing branded atorvastatin with generic versions.

## 4. Discussion

This study provides critical, real-world evidence that generic formulations yield clinical effectiveness comparable to their branded counterparts in managing secondary dyslipidemia [16]. In addition, by combining these clinical outcomes with economic modeling, this research is the first to quantify directly, at the national level in Saudi Arabia, the financial implications of switching to generic atorvastatin following the withdrawal and delisting of the branded formulation.

### 4.1. Clinical Effectiveness and Pharmacological Comparability

The use of intensive statin therapy remains central to the management of dyslipidemia and the reduction in cardiovascular risk, as stated in the latest guidelines from both the American Heart Association and the European Society of Cardiology [12,13]. In this group, both the generic and the branded form of atorvastatin were effective in lowering the main atherogenic lipid factors, namely low-density lipoprotein (LDL), triglycerides (TG), and total cholesterol (TC) (*p* < 0.0001). When the effects were adjusted for a number of other clinical variables such as patient age, existing comorbidities, the use of multiple medications, and the initial level of LDL, a multiple linear regression analysis revealed that there was no statistically significant difference in the amount of LDL reduction between the generic and the branded atorvastatin (β-estimate = −0.255, 95% CI: −0.119 to 0.391, *p* = 0.3318).

Even though this retrospective observational study was not designed with a predefined margin for a formal statistical assessment of non-inferiority, an examination of the 95% confidence interval provides useful information about the actual performance in a real-world clinical setting. The multivariable regression model yielded a 95% confidence interval of −0.119 to 0.391 for the difference in LDL reduction between the branded and generic versions. The upper bound of this interval indicates that the branded formulation might have achieved at most a 0.391% greater reduction in LDL than the generic formulation. Although it is not possible to formally claim non-inferiority, the narrow range does provide strong evidence that the two formulations are effective in everyday clinical practice. These results directly address and counter the ongoing clinical skepticism about the use of generic substitutes.

These results directly address and cast doubt on the ongoing clinical skepticism about generic substitutions. Even though there are established regulatory standards for bioequivalence, surveys show that about one-third of doctors still doubt the quality, safety, and therapeutic equivalence of generic versions [14,15]. Real-world, localized data do not back up the assertion of reduced generic efficacy. On the contrary, the findings are consistent with large-scale databases and cohort studies, all of which have shown that patient outcomes are comparable between branded and generic statins [16,17]. Earlier comparative effectiveness studies indicate that the outcome differences reported in clinical practice are largely attributable to perception bias or the “nocebo” effect rather than to genuine pharmacological inferiority [16,32].

Moreover, data from large cohort studies show that starting to take generic statin therapy leads to an 8% decrease in hospitalizations for acute coronary syndrome, stroke, or death from any cause [17]. This clinical benefit is primarily due to improved medication adherence resulting from much lower out-of-pocket expenses for patients [17,33]. Since it has been shown that the clinical lipid-lowering effects are not adversely affected, the study supports the use of generic atorvastatin as a comparable alternative.

### 4.2. National-Level Economic Impact and Health Policy

The economic burden of dyslipidemia on healthcare systems worldwide is substantial, necessitating cost-effective pharmacological interventions [6]. Although the market share of generic drugs significantly influences national drug pricing globally [19], Saudi Arabia historically maintained a lower generic market share of approximately 35% [20]. To address this, the healthcare transformation pillar of Saudi Vision 2030 led to the establishment of the National Unified Procurement Company (NUPCO) to optimize pharmaceutical procurement [20]. This strategic shift prioritized localized manufacturing and cost-effective generics [21,22], ultimately leading to the proactive withdrawal and delisting of several expensive, imported branded medications, including branded atorvastatin [23].

The study points out the considerable financial success achieved through this formulary change. Monte Carlo simulations based on 10,000 iterations indicate that generic atorvastatin yields substantial projected financial savings. From the viewpoint of the public healthcare payer, when public tender pricing is used, the estimated average annual savings amount to $29,717,778. The difference in financial terms is even greater in retail pharmacy settings, where the use of generic versions yields an average projected annual savings of $295,432,159. Although these projected savings in the retail sector represent a wider societal benefit by greatly reducing patients’ out-of-pocket costs and cutting private insurance expenditures, the tender savings directly provide policymakers with evidence that choosing generic statins reduces financial pressure on the national healthcare infrastructure [34].

### 4.3. Strengths and Limitations

A major advantage of this research is its novelty: it is the first study to examine the clinical effectiveness of generic versus branded atorvastatin and to directly measure the annual impact on the national budget following the withdrawal of the branded version from the local market. Methodologically, the study’s design is sound. To ensure a clear and accurate evaluation of the pharmacological effect, the clinical analysis used Generalized Linear Models (GLMs) to account for various covariates. Moreover, because large-scale economic impact forecasts inherently involve market uncertainty, the study addressed this issue by conducting extensive probabilistic sensitivity analyses and employing deterministic scenario matrices. This two-pronged approach provides a high degree of statistical confidence in both the clinical and economic results.

Nevertheless, the study has several limitations that should be acknowledged. Firstly, since the study is retrospective and based on a single center, the findings may not be readily applicable to the wider Saudi population or to other healthcare systems. The patients in the generic cohort were primarily treated after the formulary transition, which may introduce temporal confounding. Secondly, the fact that the cohorts were allocated in sequence, as a result of the 2019 formulary transition, also poses a risk of temporal confounding. Even though multivariable regression models were employed to account for observable differences in patient characteristics (such as baseline comorbidities and the use of multiple medications), it cannot be completely excluded that there were unmeasured changes over the decade in clinical practice, diagnostic coding, or the population’s lifestyle habits. Thirdly, although longitudinal lipid measurements were obtained, the statistical analysis depended on a baseline-to-endpoint GLM rather than a mixed-effects model. As a result, while the study confirms sustained 12-month efficacy, it does not show or compare intermediate temporal patterns of lipid reduction (for example, at 3, 6, or 9 months) between the two formulations. Fifth, since this is a retrospective chart review based on EMR data, medication adherence was assessed by using prescription renewals and pharmacy refills as proxies. Although this suggests that the medication was continuously dispensed, it cannot definitively ensure that the patient actually took it, so there remains a possibility of unmeasured secondary non-adherence. Fifth, the requirement that patients be statin-naïve was strictly based on the EMR system at our institution. Since the dataset is from a single center, some patients may have previously received statin therapy at other hospitals, private clinics, or community pharmacies, which was not recorded in our system, potentially introducing a risk of misclassifying their true historical exposure to statins. Moreover, even though the multivariable regression models successfully adjusted for observed baseline differences, such as the higher prevalence of polypharmacy and hypertension in the branded cohort, retrospective analyses remain vulnerable to residual confounding from unmeasured disease severity or specific drug–drug interactions. Also, a propensity score matching or weighting method was not used to avoid excluding unmatched cases and to maintain the minimum required sample size of 278 patients. Therefore, this non-randomized retrospective design remains prone to residual treatment-selection bias that propensity methods could otherwise have reduced. Moreover, since the final national public tender prices are not available in an open-access database, the economic simulation depended on historical institutional procurement records from KKUH. Although these price bounds were verified by internal procurement staff, the model, based on local institutional contracts, may not accurately reflect the price variations negotiated by different regional health clusters across the kingdom. Furthermore, the ten-year retrospective data-collection period (2015–2025) introduces unmeasured confounding associated with calendar time. Assignment to either the generic or branded cohort was not entirely a clinical decision; it was largely determined by changes in the institution’s formulary policies, evolving national public tender agreements, and the gradual implementation of generic substitution requirements during this period. Consequently, patients treated in later years were more likely than not to receive generic formulations. This temporal variation means that concurrent changes in clinical practice guidelines, shifts in overall disease management, or underlying secular trends in population health could contribute to residual confounding that our multivariable models cannot fully eliminate. Finally, although the economic models incorporated direct procurement costs through probabilistic sensitivity analyses, they did not consider potential indirect healthcare costs, the long-term cost-effectiveness of cardiovascular event prevention, or the complex macroeconomic relationships between increased generic market share and subsequent price deflation. Therefore, future economic evaluations should supplement pharmaceutical expenditure data with broader measures of healthcare resource utilization, including care complexity, to more accurately reflect the resources required for diverse patient populations [35]. Additionally, future real-world comparative studies could also use natural language processing to extract clinically relevant information from unstructured EHR documentation, thereby capturing treatment-related and contextual factors—which structured variables alone might miss—and possibly improving adjustment for differences between treatment groups [36].

## 5. Conclusions

The study demonstrates that generic atorvastatin is clinically comparable to the branded version in reducing low-density lipoprotein (LDL) and other atherogenic lipids over a 12-month period. If local surrogate outcomes are combined with national economic modeling, replacing branded atorvastatin with generic drugs is expected to yield large, multi-million-dollar annual savings for both public and private healthcare payers. Although prospective, multicentre studies are required to verify these findings using actual cardiovascular endpoints and comprehensive safety data, the initial results do support moving strategically towards the use of generic medications as a feasible and evidence-based method for optimizing healthcare budgets as part of the wider national healthcare transformation.

## Figures and Tables

**Table 1:** Patients’ baseline characteristics.

Characteristic	Generic (*n* = 142)	Branded (*n* = 144)	*p*-Value	Total
**Age (yr.), mean ± SD**	56.25 ± 10.18	58.06 ± 9.26	0.1169	57.16 ± 9.75
**Gender, *n* (%)**				
**Male**	60 (42.25)	61 (42.36)	0.9853	121 (42.31)
**Female**	82 (57.75)	83 (57.64)		165 (57.69)
**Body Mass Index (BMI), mean ± SD**	30.84 ± 5.91	31.26 ± 6.02	0.5425	31.06 ± 6.07
**Number of Prescription Medications, mean ± SD**	5.46 ± 2.88	6.92 ± 3.74	0.0002 *	6.20 ± 3.42
**Comorbidities, *n* (%)**				
**Diabetes**	95 (66.9)	106 (73.61)	0.2145	201 (70.28)
**Hypertension (HTN)**	69 (48.59)	87 (60.42)	0.0446 *	156 (54.55)
**Asthma**	22 (15.49)	27 (18.75)	0.4648	49 (17.13)
**Rheumatoid arthritis**	1 (0.70)	0 (0.0)	0.4965	1 (0.35)
**Chronic Kidney Disease**	2 (1.41)	8 (5.56)	0.1033	10 (3.50)
**Major Depressive Disorder**	4 (2.82)	2 (1.39)	0.4457	6 (2.10)
**Generalized Anxiety Disorder**	0 (0.0)	1 (0.69)	1.00	1 (0.35)
**Osteoporosis**	6 (4.23)	8 (5.56)	0.7853	14 (4.90)
**Osteoarthritis**	18 (12.68)	15 (10.42)	0.5499	33 (11.54)
**Number of Concurrent Active Medications**	2.01 ± 1.04	2.19 ± 1.04	0.1289	2.1 ± 1.04
**Atorvastatin Dosage, *n* (%)**				
**10 mg**	66 (46.48)	48 (33.33)	0.0012	114 (39.86)
**20 mg**	65 (45.77)	62 (43.06)		127 (44.41)
**30 mg**	0 (0.0)	1 (0.69)		1 (0.35)
**40 mg**	11 (7.75)	32 (22.22)		43 (15.03)
**80 mg**	0 (0.0)	1 (0.69)		1 (0.35)

* *p*-value < 0.05 indicating statistical significance.

**Table 2:** Lipid panel at baseline and 12-month measurement for generic and branded atorvastatin.

Variable	Baseline	Follow-Up	Mean Difference	*p*-Value
**LDL (mmol/L), mean ± SD**				
**Generic Atorvastatin**	3.46 ± 0.96	2.26 ± 0.58	−1.20 ± 0.81	<0.0001
**Branded Atorvastatin**	3.22 ± 0.96	2.36 ± 0.63	−0.86 ± 0.84	<0.0001
**TG (mmol/L), mean ± SD**				
**Generic Atorvastatin**	1.53 ± 0.70	1.33 ± 0.59	−0.20 ± 0.49	<0.0001
**Branded Atorvastatin**	1.68 ± 0.76	1.52 ± 0.63	−0.16 ± 0.55	<0.0001
**HDL (mmol/L), mean ± SD**				
**Generic Atorvastatin**	1.36 ± 0.38	1.38 ± 0.377	0.022 ± 0.236	0.7129
**Branded Atorvastatin**	1.22 ± 0.36	1.23 ± 0.332	0.006 ± 0.150	0.1864
**TC (mmol/L), mean ± SD**				
**Generic Atorvastatin**	5.51 ± 1.08	4.24 ± 0.73	−1.27 ± 0.92	<0.0001
**Branded Atorvastatin**	5.26 ± 1.07	4.31 ± 0.74	−0.95 ± 0.93	<0.0001

Note: ‘Follow-up’ values represent the lipid panel measurements recorded at the end of the 12-month continuous treatment period.

**Table 3:** Multiple linear regression to assess the impact of generic versus branded atorvastatin on LDL level.

Variable	β-Estimate	95% Confidence Limits		*p*-Value
		Lower	Upper	
**Branded vs. Generic**	−0.255	−0.119	0.391	0.3318
**Age**	−0.001	−0.008	0.005	0.6871
**Female vs. Male**	−0.052	−0.179	0.074	0.4142
**Dosage**	−0.026	−0.084	0.032	0.3814
**Number of comorbidities**	−0.045	−0.109	0.019	0.1674
**Number of Concurrent Active Medications**	0.014	−0.007	0.036	0.1890
**Baseline BMI**	0.004	−0.007	0.014	0.4710
**Baseline LDL**	−0.660	−0.726	−0.594	<0.0001

**Table 4:** Statistical Summary of Annual Costs and Projected Savings for Atorvastatin (10,000 Monte Carlo Simulations).

Variable	Mean (USD)	Median (USD)	IQR [Q1, Q3]	95% CI for Mean
**Generic Tender Cost**	13,321,182	11,581,470	[8,124,764, 16,663,940]	[13,186,441, 13,455,924]
**Generic Retail Cost**	131,826,706	110,903,303	[73,445,716, 169,301,482]	[130,274,910, 133,378,501]
**Branded Tender Cost**	43,038,960	37,521,160	[26,236,640, 53,233,936]	[42,613,772, 43,464,149]
**Branded Retail Cost**	427,258,865	371,591,099	[260,757,677, 528,239,555]	[423,011,248, 431,506,482]
**Savings (Tender)**	29,717,778	25,697,385	[17,804,068, 37,576,197]	[29,400,910, 30,034,646]
**Savings (Retail)**	295,432,159	254,334,716	[174,361,917, 374,701,008]	[292,146,122, 298,718,197]

Note: *n* = 10,000 for all variables. Savings are calculated as Branded Cost minus Generic Cost. A positive saving value indicates the generic drug is more cost-effective.

**Table 5:** Deterministic Scenario Matrix: Exact Annual Costs and Differences (Base-Case Pricing).

Total Population	Market Share	Generic Tender Cost	Branded Tender Cost	Tender Difference (Savings)	Generic Retail Cost	Branded Retail Cost	Retail Difference (Savings)
**941,105**	38.0%	$6,399,948	$20,741,418	$14,341,470	$63,578,515	$205,717,271	$142,138,756
**941,105**	52.9%	$8,909,401	$28,874,237	$19,964,836	$88,507,986	$286,380,096	$197,872,110
**941,105**	86.1%	$14,500,934	$46,995,686	$32,494,752	$144,055,530	$466,112,028	$322,056,498
**985,441**	38.0%	$6,701,453	$21,718,558	$15,017,105	$66,573,736	$215,408,731	$148,834,995
**985,441**	52.9%	$9,329,128	$30,234,519	$20,905,391	$92,677,648	$299,871,628	$207,193,980
**985,441**	86.1%	$15,184,082	$49,209,680	$34,025,598	$150,842,070	$488,070,835	$337,228,765
**1,204,428**	38.0%	$8,190,666	$26,544,907	$18,354,241	$81,367,907	$263,277,362	$181,909,455
**1,204,428**	52.9%	$11,402,269	$36,953,304	$25,551,035	$113,272,691	$366,509,801	$253,237,110
**1,204,428**	86.1%	$18,558,324	$60,145,170	$41,586,846	$184,362,547	$596,531,075	$412,168,528
**1,877,812**	38.0%	$12,769,987	$41,385,906	$28,615,919	$126,859,913	$410,473,178	$283,613,265
**1,877,812**	52.9%	$17,777,166	$57,613,538	$39,836,372	$176,602,353	$571,421,872	$394,819,519
**1,877,812**	86.1%	$28,934,103	$93,771,750	$64,837,647	$287,437,856	$930,045,807	$642,607,951

Note: “Difference” represents the total annual savings achieved by utilizing the generic formulation over the branded comparator. All figures are in USD and based on calculated base-case pricing averages.

## Data Availability

The original contributions presented in the study are included in the article; further inquiries can be directed to the corresponding author.
